# Identification of human mitochondrial RNA cleavage sites and candidate RNA processing factors

**DOI:** 10.1186/s12915-022-01373-5

**Published:** 2022-07-22

**Authors:** Guillermo Carbajosa, Aminah T. Ali, Alan Hodgkinson

**Affiliations:** Department of Medical and Molecular Genetics, School of Basic and Medical Biosciences, King’s College London, London, UK

**Keywords:** Mitochondria, RNA, QTL, Transcriptomics

## Abstract

**Background:**

The human mitochondrial genome is transcribed as long strands of RNA containing multiple genes, which require post-transcriptional cleavage and processing to release functional gene products that play vital roles in cellular energy production. Despite knowledge implicating mitochondrial post-transcriptional processes in pathologies such as cancer, cardiovascular disease and diabetes, very little is known about the way their function varies on a human population level and what drives changes in these processes to ultimately influence disease risk. Here, we develop a method to detect and quantify mitochondrial RNA cleavage events from standard RNA sequencing data and apply this approach to human whole blood data from > 1000 samples across independent cohorts.

**Results:**

We detect 54 putative mitochondrial RNA cleavage sites that not only map to known gene boundaries, short RNA ends and RNA modification sites, but also occur at internal gene positions, suggesting novel mitochondrial RNA cleavage junctions. Inferred RNA cleavage rates correlate with mitochondrial-encoded gene expression across individuals, suggesting an impact on downstream processes. Furthermore, by comparing inferred cleavage rates to nuclear genetic variation and gene expression, we implicate multiple genes in modulating mitochondrial RNA cleavage (e.g. *MRPP3*, *TBRG4* and *FASTKD5*), including a potentially novel role for *RPS19* in influencing cleavage rates at a site near to the *MTATP6*-*COX3* junction that we validate using shRNA knock down data.

**Conclusions:**

We identify novel cleavage junctions associated with mitochondrial RNA processing, as well as genes newly implicated in these processes, and detect the potential impact of variation in cleavage rates on downstream phenotypes and disease processes. These results highlight the complexity of the mitochondrial transcriptome and point to novel mechanisms through which nuclear-encoded genes can potentially influence key mitochondrial processes.

**Supplementary Information:**

The online version contains supplementary material available at 10.1186/s12915-022-01373-5.

## Background

In humans, mitochondria play important roles in many fundamental and interconnected cellular processes, such as thermogenesis, cellular energy production and cell death [1], and mitochondrial malfunction has been associated with a myriad of diverse and complex diseases such as neurodegenerative and metabolic disorders, particularly through the association of mutations in nuclear-encoded mitochondrial genes [1–5].

Mitochondria are unique organelles that contain their own independent genome, a remnant of their ancestral bacterial origin [6]. The human mitochondrial genome encodes just 2 rRNA genes, 22 tRNA genes and 13 mRNA genes, the latter coding for essential components of the OXPHOS system [7]. The compact nature of the mitochondrial genome is thought to have arisen through gene transfer to the nuclear genome over evolutionary timescales, and as a consequence, mitochondria now depend on an estimated ~1500 proteins encoded in the nucleus to carry out fundamental mitochondrial processes, including replication, transcription and translation [8, 9]. As such, both genomes coordinate to carry out metabolic processes, highlighted by the fact that the expression of numerous nuclear genes correlates with mitochondrial-encoded gene expression [10, 11].

The human mitochondrial genome itself is transcribed as polycistronic RNA containing multiple genes, which is then processed under the ‘punctuation model’ whereby tRNAs that intersperse coding and ribosomal sequences are targeted and cleaved by nuclear-encoded proteins to release gene products [12]. Canonical cleavage sites at the ends of tRNAs are processed by mitochondrial RNase P and RNase Z [13], with the cleavage of the 5′ end preceding that of the 3′ end [14]. Alongside canonical cleavage, varied processes including RNA modifications [15–17], non-canonical cleavage events [11, 18], RNA degradation [19, 20] and translation rates eventually influence the final amounts of mitochondrial proteins that will be available for use in the electron transport chain.

However, the punctuation model does not encompass all RNA cleavage events in the human mitochondria, and it is becoming clear that many other complex processes regulate the production of fully processed RNA. Furthermore, not all mitochondrial genes are flanked by tRNAs (e.g. between *MTATP6* and *MTCO3*), and thus, other proteins and mechanisms are needed to cleave RNA. For example, FASTKD family proteins have been associated with RNA processing at some gene boundaries [21]. Knock down of *FASTKD4* (*TBRG4*) has been associated with the accumulation of *ND5*-*CYB* precursors and strong reductions in mature *ND3*, *ND5* and *ATP8/6* mRNAs [22], as well as being needed for the stability of a subset of mitochondrial mRNAs [23]. Recent work has also implicated another FASTK protein, FASTKD5, at these junctions with gene knock down experiments leading to an accumulation of precursor RNAs that lack tRNA at both ends [24]. Moreover, rare non-canonical cleavage events have been observed at intra-ORFs, albeit not as frequently as at canonical processing sites, with resulting products having unknown function [11]. Similarly, regulation of these events is thought to be influenced by other factors such as RNA modifications, polyadenylation and translation factors [18, 25, 26], adding another layer of complexity. Despite this accumulation of knowledge, many genes and processes that influence the levels of fully processed mitochondrial transcripts remain unknown, and closer inspection of mitochondrial RNA cleavage events at both canonical and non-canonical junctions may allow insight into the complex regulation that occurs to influence the availability of key OXPHOS components, particularly in dynamic tissues with high energy demand. These questions become all the more pertinent, since differences in mitochondrial post-transcriptional processes have been linked to diseases such as cancer [4, 27] and hypertrophic cardiomyopathy [28].

Here, we develop a computational approach to infer and quantify human mitochondrial RNA cleavage events in standard RNA sequencing libraries by assessing the structure of RNA read placement on the mitochondrial transcriptome. We apply this approach to whole blood RNA sequencing data from over 1000 individuals to quantify variation in cleavage processes on a population scale. We identify known RNA cleavage sites at gene boundaries, but also events at non-canonical sites, that replicate in independent datasets. Comparing rates of inferred mitochondrial RNA cleavage across individuals with genetic and expression data from the nuclear genome, we identify common nuclear genetic variation in known RNA processing genes that modulate these processes across individuals (e.g. *MRPP3* and *FASTKD5*), as well as candidate genes that may play novel roles in mitochondrial RNA processing and function.

## Results

**Fig. 1:**
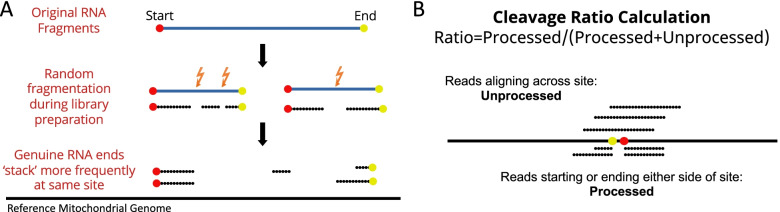
Inferred cleavage rates in mitochondrial RNA. **A** Starts and ends of original genuine RNA fragments should ‘stack’ at the same positions in the genome. **B** Cleavage ratio of a site calculated as the number of processed reads (those starting or ending either side of the site) as a proportion of total reads across the site (processed and unprocessed)

Human mitochondrial RNA is initially expressed as polycistronic transcripts that in most cases cover the whole heavy and light mtDNA strands and extensive post-transcriptional processing follows to produce individual mRNAs. Therefore, it is expected that when RNA is collected from biological samples, there will be assorted forms of precursor, intermediate and fully processed transcripts. Although many library preparation techniques also include the enrichment of polyadenylated (polyA) fragments, due to the abundance of mitochondrial RNA in any given sample, non-polyA mitochondrial RNA is also likely to be present. During the standard RNAseq library preparation protocol, this RNA is thought to be cut predominately at random positions to produce fragments of the appropriate size for sequencing. Compared to this random cleavage at internal RNA positions, we would expect that the true 5′ and 3′ ends of the original transcripts will be over-represented, and thus, more of the ends of RNA sequencing reads generated from these libraries will map to genuine cleavage sites on the mitochondrial chromosome (read stacking). Between any given pair of sites along the mitochondrial genome, there will be reads stacking immediately adjacent on either side, and by counting the number of these and then dividing this by the number of reads that fully overlap the site, we can generate a proxy ‘cleavage ratio’ for that position. Subsequently, any site that represents the start/end of a genuine transcript in the original biological sample should have a higher cleavage ratio, and by identifying these peaks, we can infer both putative RNA cleavage sites and a proxy for the rate of cleavage at these sites (Fig. 1).

### Detection and interpretation of mitochondrial RNA cleavage sites

In order to identify putative mitochondrial RNA cleavage sites, we mapped and filtered RNA sequencing datasets from 799 whole blood samples from the CARTaGENE project using parameters designed to keep as much of the genuine RNA fragment as possible (see the ‘Methods’ section) and calculated cleavage ratios at all sites across all individuals (Additional file 1: Fig. S1). We then identified positions with cleavage ratios greater than 0.1 (the mean average ratio at 100 random sites >50 bp away from known gene boundaries is 0.01, standard deviation 0.01). As many of the obtained cleavage ratios clustered close to each other along the mitochondrial transcriptome, we merged sites that were within 5bp of each other and kept the one with the highest ratio as representative of the ‘peak’ cleavage site in the region. Finally, we kept any peak site that was present in at least 50% of the 799 individuals and had at least 20x read coverage; this left us with 79 putative RNA cleavage sites (Additional file 2: Table S1). In total, 9 of these occur exactly at known gene boundaries (defined as the region between two mitochondrial genes), and a further 11 occur no further than 10bp from these regions. The remaining 59 occur at various positions within coding and non-coding sequences and may represent novel cleavage junctions. As a sanity check, we also calculated the cleavage ratios at the 28 known mRNA or rRNA gene boundaries and find that 17 are significantly higher than the background rate (*P* < 0.05/28, one-sided *t*-test, Additional file 1: Fig. S2, see the ‘Methods’ section), showing the validity of our approach.

To test the reproducibility of our cleavage detection method, we applied the approach to 344 additional whole blood RNAseq samples from the GTEx project and repeated the sample detection methods as above (for distribution of cleavage rates, see Additional file 1: Fig. S1). In total, we reproduce 54 of the original peak cleavage sites (~68%, Additional file 2: Table S1). Within these 54 reproducible sites, 8 occur exactly on known gene boundaries, all of which occur between genes that contain interspersed tRNAs, and 9 further sites were detected no more than 10 bp away from a known gene boundary. As such, our approach identifies a large number of known mitochondrial RNA cleavage sites with high accuracy. Interestingly, one reproducible cleavage site was found only 1 bp away from the *MT-ATP6* to *MT-CO3* gene boundary, which does not contain an interspersed tRNA and is thought to be processed via other mechanisms. Of the 37 reproducible sites that occur more than 10bp away from known gene boundaries, 18 occur within coding genes, 2 within rRNAs, 15 within tRNAs and 2 within the mtDNA control region. These positions are potential candidates for novel mitochondrial RNA cleavage sites.

To test the validity of the 54 reproducible sites, we performed two additional validation steps. First, both CARTaGENE and GTEx data are generated using polyA-enriched RNA fragments. Therefore, to test whether any of the 54 sites could be a result of experiment-specific technical artefacts, we obtained an additional 16 whole blood RNA sequencing datasets from Pineau et al. [29] that were generated from ribosomal RNA-depleted libraries (see the ‘Methods’ section) and tested whether the 54 putative cleavage sites had cleavage ratios >10% in any of the samples. In total, 47/54 sites were validated in these data, making it unlikely that they are a consequence of specific library preparation approaches. Second, since advances in long-read sequencing technologies now allow for the identification of full-length RNA transcripts, we tested for evidence of overlap between the 54 putative RNA cleavage sites and the 5′/3′ ends of RNA fragments generated from high coverage Oxford Nanopore cDNA and native RNA sequencing data from a human B lymphocyte cell line [30]. Within this study, it was shown that technical features of Nanopore direct RNA sequencing led to truncation events in mitochondrial transcripts 10–15bp from the 5′ end of genes, and as such, we removed putative cleavage sites within 3bp of these regions. Of the 44 putative cleavage sites remaining, we find evidence for validation of 29 sites (see the ‘Methods’ section). As such, in total, we validate 52/54 putative cleavage sites across ribosomal RNA-depleted and Nanopore datasets (Additional file 2: Table S1; for flowchart of filtering steps, see Additional file 1: Fig. S3). Although some of the 54 putative RNA cleavage sites may still be false positives or not validated because of technical features specific to each platform, since we are also interested in the molecular mechanisms underpinning these events, as well as the potential downstream consequences of variation in such processes, we continue to focus on these 54 sites for all subsequent analyses.

It has been shown in previous work that reads also tend to terminate and ‘stack’ at sites of RNA modification [31–33], and indeed, in our data, five putative RNA cleavage sites occur at known modified sites. These sites include position 5595 (an m^1^A modification at position 9 of *MT-TA*), position 7465 (a dihydrouridine modification at position 20 of *MT-TS1*), position 14699 (a m^2^G modification at position 26 of *MT-TE*) and positions 7544 and 8322 (Ψ modifications at positions 27 and 28 of *MT-TD* and *TK*, respectively). Furthermore, work by Mercer et al. [11] found evidence of additional RNA cleavage events within the mitochondrial transcriptome that generate short RNAs (sRNAs) that have unknown function, but may play a role in RNA silencing. Three cleavage sites detected in this study occur close to identified sRNA boundaries: one at site 598 within *MT-TF* (1bp from an sRNA boundary), the second at site 3258 within *MT-TL1* (0bp from an sRNA boundary) and the third at site 9157 within *MT-ATP6* (1bp from an sRNA boundary). Although stringent criteria were used to identify such sRNA boundaries, these results show that our approach has the potential to identify novel RNA cleavage events with functional relevance.

To test whether RNA cleavage events either influence or co-occur with downstream processes, we compared cleavage ratios to mitochondrial-encoded gene expression levels across individuals, requiring significance (after Bonferroni correction) and the same direction of effect in both discovery (CARTaGENE) and replication (GTEx) datasets. In total, we find 18 significant relationships, involving 6 unique cleavage sites (positions 659, 1682, 9219, 10074, 10479 and 10496) and the expression levels of 9 unique mitochondrial genes (Additional file 2: Table S2 and Additional file 1: Fig. S4). Position 1682 falls 12bp from the 5′ end of *MT-RNR2*, and putative cleavage ratios at this site are negatively associated with the expression levels of nine different mitochondrial genes (*MT-RNR1*, *RNR2*, *ND1*, *ND2*, *CO2*, *ATP6*, *ND4*, *ND5* and *CYB*). This may suggest a role for cleavage at position 1682 in modifying the processing and expression of *MT-RNR2*, which subsequently influences the RNA levels of most other mitochondrial-encoded genes, although this would need to be tested further. The remaining 8 cleavage sites are all associated with the expression levels of four genes (*MT-RNR1*, *RNR2*, *ND1* and *ND2*).

### Quantitative trait loci mapping

Post-transcriptional processing of the mitochondrial transcriptome is carried out exclusively by nuclear-encoded proteins. Therefore, in order to identify common nuclear genetic variants and genes associated with variation in mitochondrial RNA cleavage events across individuals, we used genome-wide genetic data and the cleavage ratio at each of the 54 cleavage sites for 799 individuals in the CARTaGENE dataset to map quantitative trait loci in the nuclear genome.

**Fig. 2:**
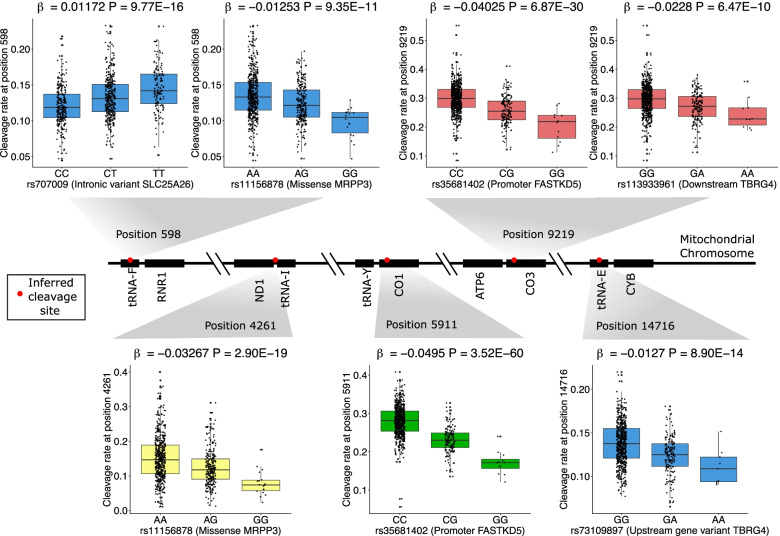
Relationship between genotype and inferred cleavage rate at multiple positions on the nuclear genome and mitochondrial transcriptome, respectively. Inferred cleavage positions are represented by red circles along the mitochondrial transcriptome, and inferred cleavage ratios are colour coded using three categories: yellow on known gene boundaries, blue within tRNAs, green on mRNAs within a tRNA flanked boundary and red on mRNA within a non-tRNA flanked boundary. Beta estimates, *P*-values, cleavage rates and genotypes displayed in boxplots originate from the CARTaGENE dataset (*N* = 799 for each plot)

In total, we identify 26 nuclear genetic variants associated with mitochondrial RNA cleavage rates (unique peak nuclear genetic variant and cleavage site pairs) after correcting for multiple tests (Table 1, *P* < 9.26 × 10^−10^, correcting for standard genome-wide significance at 5 × 10^−8^ at 54 sites, examples shown in Fig. 2, for QQ plots see Additional file 1: Fig. S5). These 26 nuclear-encoded variants are associated with 24 different cleavage sites in the mitochondrial transcriptome; 7 of these fall within 2bp of known gene boundaries (between *MTND1-TRNI*, *MTND2-TRNW*, *TRNS1-TRND*, *MTATP6-MTCO3*, *MTCO3-TRNG*, *MTND3-TRNR* and *MTND4-TRNH*), 12 fall within a tRNA (ranging from 10 to 31bp from either end) and 5 fall within coding regions (10bp from the 3′ end of *MTND1*, 8bp from 5′ end of *MTCO1*, 13bp from 3′ end of *MTATP6*, 12bp from 5′ end of *MTCO3* and 12bp from 5′ end of *MTCYB*).

**Table 1:** Nuclear-encoded genetic variation significantly associated with inferred mitochondrial RNA cleavage rates in discovery (CARTaGENE) data and statistics for the same associations in replication (GTEx) data. Variants are linked to genes either through functional (missense) mutations or via eQTL data

Cleavage Site	Peak nuclear SNP	Chr	Position	Annotation	eQTL	Nearest nuclear gene	CaG beta	CaG ***P***-value	GTEx beta	GTEx ***P***-value
598	rs707009	3	66372144	Intron/NMD	LIPA, LRIG1, SIRT1, SLC25A26	SLC25A26	0.012	9.8E−16	−0.011	2.8E−02
598	rs11156878	14	35735967	Missense	NA	MRPP3	−0.013	9.4E−11	−0.011	1.2E−01
3246	rs74422990	14	35726093	Intron	MRPP3, PPP2R3C	MRPP3	−0.036	4.3E−28	−0.028	3.7E−04
3258	rs11156878	14	35735967	Missense	NA	MRPP3	−0.010	4.3E−18	−0.004	1.5E−01
4252	rs74422990	14	35726093	Intron	MRPP3, PPP2R3C	MRPP3	−0.034	1.9E−14	−0.032	4.3E−05
4261	rs11156878	14	35735967	Missense	NA	MRPP3	−0.033	2.9E−19	−0.045	5.4E−04
4411	rs74422990	14	35726093	Intron	MRPP3, PPP2R3C	MRPP3	−0.064	1.0E−35	−0.078	2.5E−09
4438	rs11156878	14	35735967	Missense	NA	MRPP3	−0.013	1.2E−17	−0.002	6.6E−01
5511	rs11156878	14	35735967	Missense	NA	MRPP3	−0.037	3.2E−11	−0.014	2.4E−01
5911	rs35681402	20	3138626	Intron	ITPA, UBOX5, PROSAPIP1, FASTKD5	UBOX5/FASTKD5	−0.050	3.5E−60	−0.058	5.1E−07
7514	rs74422990	14	35726093	Intron	MRPP3, PPP2R3C	MRPP3	−0.041	7.9E−13	−0.015	3.3E−01
7543	rs7158706	14	35736692	Intron	MRPP3	MRPP3	−0.022	2.2E−17	−0.033	2.0E−03
8321	rs74422990	14	35726093	Intron	MRPP3, PPP2R3C	MRPP3	−0.019	5.4E−18	−0.005	5.3E−01
9194	rs75286457	20	3138134	Intron	ITPA, UBOX5, PROSAPIP1, FASTKD5	UBOX5/FASTKD5	−0.016	8.6E−23	−0.014	1.7E−02
9205	rs75286457	20	3138134	Intron	ITPA, UBOX5, PROSAPIP1, FASTKD5	UBOX5/FASTKD5	−0.031	8.0E−17	−0.043	1.7E−03
9219	rs35681402	20	3138626	Intron	ITPA, UBOX5, PROSAPIP1, FASTKD5	UBOX5/FASTKD5	−0.040	6.9E−30	−0.042	1.6E−04
9219	rs113933961	7	45051924	Intron	CCM2, RP4-647J21.1, MYO1G, SNHG15, NACAD, TBRG4, H2AFV	CCM2/TBRG4	−0.023	6.5E−10	0.012	1.7E−01
9990	rs11156878	14	35735967	Missense	NA	MRPP3	−0.037	4.2E−11	−0.015	1.4E−01
10404	rs11156878	14	35735967	Missense	NA	MRPP3	−0.039	8.1E−11	−0.030	4.1E−03
10432	rs11156878	14	35735967	Missense	NA	MRPP3	−0.039	1.2E−24	−0.006	5.4E−01
12137	rs11156878	14	35735967	Missense	NA	MRPP3	−0.033	5.0E−11	−0.020	2.4E−02
12280	rs11156878	14	35735967	Missense	NA	MRPP3	−0.019	2.5E−16	−0.015	6.2E−02
14698	rs113933961	7	45051924	Intron	CCM2, RP4-647J21.1, MYO1G, SNHG15, NACAD, TBRG4, H2AFV	CCM2/TBRG4	−0.009	5.8E−10	0.001	7.3E−01
14716	rs73109897	7	45156242	Upstream gene	CCM2, RP4-647J21.1, MYO1G, SNHG15, NACAD, TBRG4, H2AFV	TBRG4	−0.013	8.9E−14	−0.007	6.5E−02
14758	rs4724362	7	45161303	Intergenic	CCM2, RP4-647J21.1, MYO1G, SNHG15, NACAD, TBRG4, H2AFV	TBRG4	−0.069	1.7E−10	−0.013	2.7E−01
15968	rs61988271	14	35741554	Intron	MRPP3, SNX6	MRPP3	−0.024	3.6E−10	−0.026	3.5E−02

To identify the potential nuclear genes that are modulating mitochondrial RNA cleavage rates, we tested whether each significant peak nuclear variant was either functional or associated with the expression of a nearby gene in the eQTLGen consortium database [34] (*P* < 5 × 10^−8^, selecting the nearest gene if multiple associations were found). Applying this approach, we link a number of known and novel proteins involved in mitochondrial RNA cleavage (Table 1). First, a large number of peak nuclear genetic variants are missense and intronic mutations linked with *MRPP3*, and in all cases, these associations occur for mitochondrial cleavage sites that fall at mRNA-tRNA boundaries, or within a mitochondrial tRNA. MRPP3 is known to cleave the 5′ end of mitochondrial tRNAs at canonical mRNA-tRNA junctions, but results here suggest the gene may also cleave internal tRNA positions that could result in short RNA fragments that are generated from the ends of tRNAs (described above). Second, several peak nuclear genetic variants are intronic mutations linked with *FASTKD5* and are associated with mitochondrial RNA cleavage events near to the *MTATP6-MTCO3* junction, as well as a site near to the 5′ end of *MTCO1*. FASTKD5 has been shown to be required for the maturation of precursor mitochondrial RNAs that are not flanked by tRNAs [24] and results here therefore validate this finding. Third, multiple peak nuclear variants are intronic or upstream mutations for *TBRG4* (*FASTKD4*), which are associated with mitochondrial RNA cleavage specifically around the *MT-TE-MTCYB* junction, but also for a cleavage site close to the *MTATP6-MTCO3* junction that is not interspersed by a tRNA. TBRG4 is known to play a role in processing mitochondrial RNA precursors, as well as stabilising several mitochondrial mRNAs, but these results hint that TBRG4 may also be involved in the processing of the non-canonical junction between *MTATP6-MTCO3*. Fourth, an intronic mutation within *SLC25A26* is associated with cleavage rates within *MT-TF*. SLC25A26 is a mitochondrial carrier protein involved in transporting S-adenosylmethionine into the mitochondria [35, 36]. We have previously implicated genetic variants in this protein with variation in mitochondrial RNA modification levels [26, 37], and thus, the link we observe here may be modulated through this process.

In order to test the robustness of our findings, we attempted to replicate significant associations in an independent whole blood dataset (GTEx, using the same peak variant where present, or the closest variant in high LD, *R*^2^ > 0.9, if not). In total, 11 of the 26 peak nuclear genetic variants show the same direction of effect with the same high-confidence cleavage site in GTEx data at nominal significance (*P* < 0.05), 7 of which are significant after Bonferroni correction (Table 1, *P* < 0.00192, corrected for 26 tests). Furthermore, association betas show strong correlation between datasets (Pearson’s *R* = 0.623, *P* = 0.0002, Additional file 1: Fig. S6). Associations that replicate include a missense mutation in *MRPP3* that is linked to a cleavage event that is exactly at the *MTND1-MTTI* gene boundary, as well as links between intronic *FASTKD5* mutations and cleavage rates at sites near the *MTATP6-MTCO3* and the *MTY-MTCO1* junctions, thus validating the role of FASTKD5 in these processes.

### Mitochondrial RNA cleavage events and nuclear-encoded gene expression

Given the role of nuclear-encoded proteins in mitochondrial RNA processing events, we sought to further explore these complex cross-genome relationships by directly comparing inferred mitochondrial RNA cleavage ratios with nuclear-encoded gene expression in the same individuals. Given the influence of multiple interconnected genetic and environmental factors on variable gene expression, we implemented a stringent set of filtering strategies in order to identify nuclear genes that may be modulating mitochondrial RNA cleavage events.

First, using linear regression, we identified nuclear genes whose expression was associated with inferred mitochondrial RNA cleavage rates at the 54 reproducible sites in both discovery (CARTaGENE) and replication (GTEx) datasets (applying Bonferroni correction for the number of sites and the number of genes, for pairs that were present in both datasets). This approach identified 14,414 gene-site pairs in the discovery dataset (see Additional file 2: Table S3 for all significant associations) and 465 in the replication dataset (see Additional file 2: Table S4 for all significant associations, and Additional file 1: Fig. S7 for *P*-value distributions from both the discovery and replication data). We then intersected the two lists, keeping only those associations with the same direction of effect, which left 52 gene-site pairs encompassing 43 unique genes (Additional file 2: Table S5). Five of these genes are present in MitoCarta [8] and another five are thought to be RNA binding proteins [38] (non-overlapping sets, except *COX5B*). To test whether the relationship between each nuclear gene/mitochondrial RNA cleavage site pair is more likely to be driven by the nuclear gene (rather than caused by mitochondrial RNA processes), we performed mediation analysis by identifying significant peak *cis*-eQTLs in the nuclear genome for each of the 43 unique genes and testing whether these variants are first associated with the cleavage ratio of the corresponding mitochondrial site (*P* < 0.05) and second whether this relationship is significantly mediated by the expression of the nuclear gene (*P* < 0.05/52). In total, 12 of the tests show significant evidence for mediation; 3 of these are for site 1682 in the mitochondrial genome, which falls within *MTRNR2* close to a tRNA junction; 2 are for site 9157, which is closest to the *MTATP6-MTCOX3* junction; and the remaining 7 are for site 10074, which falls within *MTND3* near to a tRNA junction. The 10 unique genes identified through this analysis are thus candidates for being involved in mitochondrial RNA cleavage. They include *ATP5E* and *COX17*, both of which form part of the electron transport chain, as well as *CXCR2P1*, *ELOVL7*, *GNAZ*, *ITGB5*, *MAP3K7CL*, *MYLK*, *SH3BGRL2* and *TUBB1*.

Finally, to test whether nuclear genes might be operating through mitochondrial RNA cleavage to influence mitochondrial-encoded gene expression levels, we took all mitochondrial RNA cleavage sites that were significantly associated with both the expression of a nuclear-encoded gene and the expression of a mitochondrial-encoded gene (125 unique cases) and performed a further round of mediation analysis. In each of these cases, we first tested whether the expression of the nuclear- and mitochondrial-encoded genes were correlated (*P* < 0.05) and then tested whether this relationship is significantly mediated by the inferred cleavage rate of the associated site (*P* < 0.05/125). In total, 16 of the tests show significant evidence for mediation, implicating 9 unique nuclear genes (*ACRBP*, *CTTN*, *CXCR2P1*, *GNAZ*, *ITGB5*, *MAP3K7CL*, *SH3BGRL2*, *SPARC* and *TMEM40*, Additional file 2: Table S6). None of these genes are present in MitoCarta [8], and as such, they may have unidentified roles in mitochondrial processes, either directly or indirectly through interactions with other genes.

### Knock down of candidate novel mitochondrial RNA processing genes

**Fig. 3:**
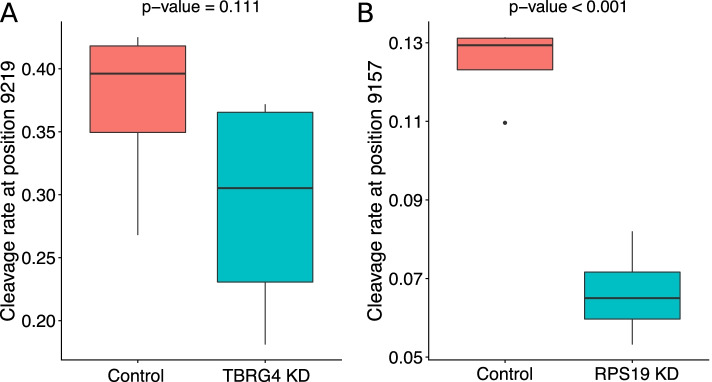
Comparison of inferred cleavage rates in mitochondrial RNA between control and shRNA knock down data for candidate genes (*N* = 8 in each plot, 4 from KD and 4 from controls). **A** Control vs sh knock down cleavage ratio for TBRG4 at position 9219 near the *MT-ATP6* to *MT*-*CO3* gene boundary. **B** Control vs sh knock down cleavage ratio for RPS19 at position 9157 near the *MT-ATP6* to *MT*-*CO3* gene boundary

In order to further implicate nuclear-encoded genes in mitochondrial RNA processing, we sought knock down (KD) data for any gene that has been implicated above in quantitative trait loci mapping (four unique genes — *MRPP3*, *FASTKD5*, *TBRG4* and *SLC25A26*) and expression correlation analyses (43 unique genes, Supplementary Table 5). In total, two of these genes (*TBRG4* and *RPS19*) have shRNA KD data from the ENCODE project, containing 8 samples in total (4 from KD and 4 from controls) in 2 different cell lines. Using these data, we mapped and filtered samples as above and calculated cleavage ratios at mitochondrial RNA cleavage sites linked to the discovery of each gene. First, *TBRG4* (*FASTKD4*) has been implicated in influencing cleavage ratios around the *MTATP6-MTCOX3* junction that is not separated by a tRNA. Using KD data for this gene, we find that there is a decrease in the cleavage ratio in KD samples compared to controls at position 9219 (mean ratio 0.37 for control and 0.29 for KD samples), although this is not significant (*P*=0.111, one-tailed *t*-test, Fig. 3). The closest high-confidence site to the exact junction between *MTATP6* and *MTCOX3* that we detect falls at position 9207, and ratios at this site in KD samples are again lower than in control data, but this difference is also not significant (*P* = 0.295).

Second, *RPS19* is a nuclear-encoded ribosomal gene whose expression is associated with putative cleavage ratios at site 9157, in *MTATP6*, 49bp from the *MTATP6-MTCOX3* junction. Using KD data for this gene, we find that there is a highly significant decrease in the cleavage ratio in KD samples compared to controls (*P* = 0.00018, one-tailed *t*-test, mean control ratio = 0.125, mean KD ratio = 0.066, Fig. 3), suggesting that RPS19 may be modulating RNA processing at this site. To test whether RPS19 may be acting more globally across the mitochondrial transcriptome, we tested for differences between control and KD data for all 54 reproducible cleavage sites and find that no other sites are significant after Bonferroni correction, and indeed, the relationship at site 9157 is the only one significant at this level (*P* < 0.05/54). Collectively, these results implicate a novel gene (*RPS19*) in modulating mitochondrial RNA cleavage events.

### Functional enrichment and potential disease links

In order to identify whether mitochondrial RNA cleavage events might be linked to disease, we first tested whether any peak nuclear genetic variants associated with mitochondrial RNA cleavage rates (identified above) were in linkage disequilibrium (LD) with variants listed in the GWAS catalogue [39] (*R*^2^ > 0.8 in the CEU population from 1000 Genomes data [40], disease associations *P* < 5 × 10^−8^). In doing so, we find that both rs4724362 and rs73109897 (which both appear to act through *TBRG4* on sites around the *MT-TE* and *MT-CYB* junction) are in LD with rs12672022, which is associated with colorectal cancer.

Second, we tested for functional enrichment in GO and KEGG terms for nuclear genes whose expression correlated with mitochondrial RNA cleavage rates across both discovery and replication cohorts (43 unique genes) using gProfiler [41]. After adjusting for multiple tests, no GO terms were significantly enriched for the gene list; however, several KEGG pathways were enriched including oxidative phosphorylation (4 genes, adjusted *P* = 0.016), but also amyotrophic lateral sclerosis, (6 genes, adjusted *P* = 0.012), Parkinson’s disease (5 genes, adjusted *P* = 0.019), Prion disease (5 genes, adjusted *P* = 0.028), Huntington’s disease (5 genes, adjusted *P* = 0.040) and pathways of neurodegeneration — multiple genes (6 genes, adjusted *P* = 0.039).

## Discussion

Due to the polycistronic nature of the transcription of the human mitochondrial genome, post-transcriptional events are particularly important for determining downstream events. Despite this, the genetic and molecular mechanisms modulating variation in these processes across individuals remain poorly understood. In order to elucidate key mitochondrial RNA processing events, we developed an approach to identify putative RNA cleavage sites and rates using standard RNA sequencing data. In doing so, we find 54 mitochondrial RNA cleavage junctions that are reproducible across independent whole blood datasets. Many of these sites align with well-known cleavage boundaries, thus validating our approach, but a substantial fraction also occur at novel sites, opening up the possibility of new mechanisms by which the mitochondrial transcriptome is regulated.

There are several potential limitations to our approach. First, discovery of putative human mitochondrial RNA cleavage sites occurs in RNA sequencing data that has been enriched for polyadenylated RNA. Not all mitochondrial transcripts are polyadenylated [18], and therefore, this RNA preparation step will likely lead to biases in the mitochondrial RNA fragments that are sequenced. However, due to the highly abundant nature of mitochondrial RNA in cells, we observed good coverage of the entire mitochondrial genome in these datasets (e.g. in CARTaGENE samples, 99.5% of all sites across all samples have >100X coverage), suggesting that many mitochondrial-encoded transcripts are well represented. Second, fragmentation of RNA does not always occur randomly during library preparation, with known biases occurring in AT-rich regions for example. Such biases could lead to artefacts in our data that are reproducible across experiments using the same methods. We attempt to alleviate these effects by testing for evidence of replication of putative RNA cleavage sites in alternative datasets, finding that almost all are present either in independent Oxford Nanopore sequencing data or in sequencing data generated using material that has been depleted of ribosomal RNA. Third, ‘stacking’ of RNA sequencing reads at the starts and ends of genuine RNA fragments tends to show a more gradual decline around known gene boundaries, rather than a clean signal. Although this makes the exact locations of putative RNA cleavage sites more difficult to detect, we attempt to reduce this problem by identifying the strongest signal of cleavage in the local region (see the ‘Methods’ section). However, since Oxford Nanopore sequencing data also contains prematurely truncated transcripts [30], despite our efforts to focus only on the most abundant transcript terminal sites (see the ‘Methods’ section), it cannot be ruled out that some of the putative RNA cleavage sites that are validated in these data are a consequence of this technical phenomenon. Fourth, it has previously been suggested that aberrant, partially digested mitochondrial RNAs undergo polyadenylation in humans to promote degradation [42], which could be observed in our data as putative RNA cleavage sites. However, analysis of sequences with intra-gene polyadenylation showed that not only were they reasonably rare events compared to full-length polyadenylated transcripts, but also that the 3′ end of polyadenylated sequences was dispersed throughout each gene and not clustered [42]. As such, it seems unlikely that they would ‘stack’ at the same sites across the majority individuals, as we observe here.

The putative cleavage sites detected fall across many different regions of the mitochondrial genome, including at or close to known gene boundaries, or directly within different tRNAs, rRNAs or mRNAs. By comparing inferred RNA cleavage rates to mitochondrial-encoded gene expression levels, we see a number of strong relationships that could have important implications for mitochondrial function. Within this, 10 of the sites fall within coding regions and are far away from known gene boundaries (>20bp). These sites are unlikely to be artefacts driven by alternative post-transcriptional events such as RNA edits (since they do not overlap known edit sites [37]) or RNA modifications (see the ‘Results’ section) and may be particularly interesting as they may modulate mRNA levels directly. Indeed, 3 of these sites show significant associations with mitochondrial-encoded gene expression levels in both the discovery and replication datasets. Disentangling the direct downstream functional consequences of novel mitochondrial RNA cleavage sites more generally will require further experimental work.

Cleavage of human mitochondrial RNA at gene boundaries is known to be carried out by the RNase P (MRPP1, MRPP2 and MRPP3) [13, 14, 43] and Z enzymes (ELAC2) [44], as well as at least one FASTKD protein (FASTKD5) [24], yet the full compendium of genes involved in these processes is yet to be discovered. Using inferred mitochondrial RNA cleavage ratios, we link a number of nuclear-encoded genes to mitochondrial RNA processing through quantitative trait loci mapping. These include genes already implicated in RNA cleavage described above (*MRPP3* and *FASTKD5*), but also *FASTKD4* (*TBRG4*) and *SLC25A26*. Knock down of *FASTKD4* has been shown to influence expression levels of *MTATP6* and *MTCO3* [22, 23], which are not separated by a tRNA and therefore are not processed in the same way as the majority of mitochondrial-encoded mRNAs; however, our results here suggest that the gene may be directly involved in RNA cleavage around the *MTATP6-MTCO3* junction. SLC25A26 has previously been linked with mitochondrial RNA modification levels, consistent with its role as a S-adenosylmethionine transporter; therefore, it seems likely that the association we find here is modulated through the gene’s effects on RNA modification.

We also further implicate nuclear-encoded genes in human mitochondrial RNA processing by comparing inferred mitochondrial RNA cleavage ratios to nuclear-encoded gene expression. In doing so, we find 43 unique genes that show strong relationships with these processes across independent datasets. Within these, we use mediation analysis to show that ten genes are possibly acting in a causal manner, rather than in response to changes in mitochondrial gene expression. These genes include two nuclear-encoded electron transport chain proteins (ATP5E and COX17) that may be acting directly on RNA, but are more likely to be triggering changes in mitochondrial RNA processing through intermediate mechanisms. The remaining eight are strong candidates for involvement in mitochondrial RNA cleavage events that could be followed up with further functional work.

We validate some of our findings by integrating gene knockdown data from the ENCODE project and find that the expression of *RPS19* is not only associated with cleavage rates at site 9157 (49bp from the *MTATP6-MTCOX3* junction) in two independent RNA sequencing datasets, but shRNA knock down of the gene in HepG2 and K562 cells causes highly significant changes in the RNA cleavage ratios at the same site. *RPS19* is a nuclear-encoded ribosomal gene containing an RNA binding domain [45]. Although *RPS19* is not listed in MitoCarta, it is predicted to have a mitochondrial targeting peptide in iPSORT [46]. This may suggest that RPS19 is directly involved in cleaving mitochondrial RNA; however, it also remains possible that the protein indirectly modulates other processes that influence mitochondrial RNA post-transcriptional processes.

Finally, it is possible that human mitochondrial RNA cleavage events play a role in cell function and disease. Previous work has shown that knock down of key mitochondrial RNA binding proteins in mice leads to phenotypes such as obesity [47], cardiomyopathy [14, 48–50] and premature death [48], therefore linking post-transcriptional processes in mitochondrial to some of the most common human complex diseases. As such, the novel genes we identify here may be good candidates for playing roles in disorders linked to mitochondria. Indeed, we see some evidence of this as genetic variants associated with mitochondrial RNA cleavage rates are in LD with those associated with colorectal cancer. Similarly, nuclear genes whose expression correlates with mitochondrial RNA cleavage rates are enriched for those linked to Parkinson’s disease, amyotrophic lateral sclerosis, Prion disease and Huntington’s disease. Within this, for genes where we infer the causal direction of association through mediation analysis, *ATP5E* (a component of the electron transport chain) has been linked to mitochondrial ATP synthase deficiency [51], *ELOVL7* (a fatty acid elongase) has recently been associated with Parkinson’s disease [52] and other brain-related traits [53], and *ITGB5* (an integrin subunit) has been associated with blood pressure [54], a clinically relevant trait that we have previously found to be linked to mitochondrial processes [25, 26]. It will therefore be intriguing to further explore these genes in a functional setting.

## Conclusions

In summary, our work interrogates large quantities of existing RNA sequencing data using novel approaches to identify putative RNA cleavage sites in mitochondrial RNA. We also use inferred cleavage rates at these sites within QTL and expression cross-correlation analyses to highlight nuclear-encoded genes that potentially influence important mitochondrial post-transcriptional processes. We validate the link between one of these genes, *RPS19*, and inferred mitochondrial RNA cleavage rates using gene knock down data, and more generally, it will be interesting to interrogate the roles of these genes in mitochondrial function. Since mitochondrial DNA is largely transcribed as polycistronic strands of RNA, identifying post-transcriptional events that influence the expression of key elements of the electron transport chain could lead to valuable insights across multiple strands of fundamental and disease biology.

## Methods

### Data description

RNA sequence and genotype data were obtained from two independent, publicly available projects:

CARTaGENE [55]: CARTaGENE is a population-based cohort of healthy individuals aged 40–69, from Quebec, Canada. Whole blood samples were obtained for RNA sequencing and genotyping, generating 100-bp paired-end RNAseq reads and genotypes from the Illumina Omni2.5M genotyping array for 911 individuals. Samples with RNAseq data from multiple sequencing runs were merged before being aligned.

GTEx (Genotype-Tissue Expression) Project [56]: Samples were collected from 354 deceased individuals for RNA sequence analysis and dense genotyping. We used data from both the pilot and midpoint phases of the GTEx project, where samples were genotyped in the Illumina Omni5M and Illumina Omni2.5M genotyping arrays, respectively. RNAseq reads produced by the project varied in length, and we used only samples with 75-bp long reads.

### RNA sequencing alignment and cleavage site inference

For each sample, RNAseq reads were trimmed to remove adapter sequences using TrimGalore [v0.4.0] using a stringency parameter value of 3 (www.bioinformatics.babraham.ac.uk/projects/trim_galore) and Poly-A/T sequences >5 bp using PRINSEQ-lite [v0.20.4] [57]. No quality trimming was performed in order to maintain the genuine RNA fragment end. Remaining reads with >20 nucleotides were then mapped to the human reference sequence (1000G GRCh37 reference, which contains the mitochondrial rCRS NC_012920.1) using STAR [2.6.1d] [58] with the EndToEnd alignEndsType flag (again, to avoid read trimming). SAMtools [v1.4.1] [59] was then used to retain only properly paired and uniquely mapped reads. Read start and end positions were then identified with SAMtools (start positions defined as the value of the POS field, 4th column, and end positions as POS+TLEN-1) and used to calculate the cleavage ratios as above.

To assess cleavage ratios at gene boundaries, we considered a region within 3bp of each GENCODE (v19) annotated boundary for ribosomal and messenger RNA mitochondrial genes. Within each region, we then identified the position with the highest cleavage ratio as representative of the gene boundary and then obtained the distribution of cleavage ratios for this site across all individuals. For the background rate, we randomly selected 100 sites from locations at least 50bp away from a gene boundary region and followed the procedure as above for each site. We used one-sided *t*-tests to assess if the gene boundary cleavage ratio was higher than the background rate and applied Bonferroni correction to account for the 28 gene boundaries tested.

To validate mitochondrial RNA cleavage sites using data generated with other library preparation and/or sequencing techniques, we first obtained an additional 16 whole blood RNA sequencing datasets from healthy controls, generated after ribosomal RNA depletion (rather than polyA enrichment) and sequenced on the Illumina Hiseq 4000 platform [29] (GEO accession GSE136371). For each sample, we aligned data and generated RNA cleavage ratios at each site as above and then tested whether any sample had a cleavage ratio of >10% at any site within 3bp of each of the 54 putative RNA cleavage sites identified in CARTaGENE and GTEx data. Next, we obtained publicly available aligned native RNA and cDNA sequencing of NA12878, generated on the Oxford Nanopore MinIon by the Nanopore WGS consortium [30] (https://github.com/nanopore-wgs-consortium/NA12878). Within this study, data was aligned with minimap2 to the GRCh38 human genome reference, which contains the exact same mitochondrial sequence as the reference used here (1000G GRCh37 reference) and data was merged across all sequencing runs to create a single alignment file for each of the native RNA and cDNA data. For each alignment file, we extracted sequencing reads that mapped to the mitochondrial genome, were labelled as the primary alignment and had mapping quality greater than 30, and then removed reads that had segments that aligned elsewhere in the mitochondrial genome and those that aligned elsewhere in the nuclear genome with equal or greater mapping quality score. For the remaining data, we calculated the start and end positions of each read using the CIGAR string (which contains information on sequence matches and insertions/deletions for each read versus the reference sequence). To test for an overlap with putative RNA cleavage sites identified in short read data, we identified start and end positions in long read data that had at least 200 supporting reads and were in the top 1/50 of sites across the mitochondrial genome in terms of the number of reads that started or ended at that position. For validation, we required these positions to be within 3bp of the putative RNA cleavage site.

### Quality control, phasing and imputation of genotype data

QTLtools [v1.0] (https://qtltools.github.io/qtltools/) was used to check for consistent sample labelling between genotype and RNAseq data. Quality control was carried out using PLINK [v1.90b3.44] [60], removing duplicate samples, those with unexpected relatedness, genetic PC outliers and samples with outlying heterozygosity rates. We also removed samples with discrepant reported and genotypic sex information, those with >5% missing data and those with ambiguous X chromosome homozygosity estimates. SNPs were filtered for violating Hardy–Weinberg equilibrium (HWE) (*P* < 0.001), for having a minor allele frequency (MAF) < 1% or for having a genotype missingness >5%. SNPs coded according to the negative strand were flipped to the positive strand. SNPs remaining on autosomal chromosomes were phased using default settings within SHAPEIT [v2.r837] [61].

Phased chromosomes were imputed using IMPUTE2 [v2.3.2] [62, 63] using 1000 Genomes Phase 3 individuals as a reference population and default settings in 2 Mb intervals. Imputed genotypes were then hard called using GTOOL [v0.7.5] (www.well.ox.ac.uk/~cfreeman/software/gwas/gtool.html) with a minimum calling threshold of 0.9 and then filtered for having an IMPUTE2 info score <0.8, genotype missingness > 5%, MAF < 5%, HWE P < 0.001 or for being multi-allelic. GTEx data, which was genotyped on two different arrays, were imputed separately and then merged.

### Quantitative trait loci mapping

Quantitative trait loci mapping was carried out for the 54 reproducible cleavage sites identified in both CARTaGENE and GTEx datasets. Analyses were carried out separately for each position (therefore comparing samples that were generated using the same library preparation and sequencing protocols), using linear models in PLINK [v1.9]. Covariates used in the linear model included 5 study-specific genetic PCs and 10 PEER factors calculated from RNAseq data using PEER [v1.0] [64]. PEER factors were calculated per dataset using all genes (nuclear and mitochondrial) that had a mean TPM >2, including no covariates. Additional covariates included in the linear model were sex and RNA sequencing batch information, where available and where relevant.

### Nuclear gene expression linear regression

Mitochondrial and nuclear gene expression were generated as in [25]. The lm function within R was used to regress the inferred cleavage ratios against nuclear and mitochondrial-encoded gene expression levels, including 10 PEER factors as covariates, in CARTaGENE and GTEx independently. The obtained regression *P*-value was adjusted for Bonferroni correction using the p.adjust function and the two lists of significant associations we intersected (requiring significance in the discovery and replication datasets, with the same direction of effect), identifying 52 significant associations (47 of which remained significant under the same criteria after also including the first 5 genetic principle components in each linear model). Since the discovery dataset identified a significantly larger number of associations (14,414) when compared to the replication dataset (465), we checked the influence of sample size on these results by randomly resampling the discovery dataset without replacement down to the same size as the replication set (*n* = 344) and repeating the analysis as above. In doing so, we find 2259 significant associations (after Bonferroni correction), which more closely matches the number of associations found in the replication dataset. The remaining differences may be driven by random variation between the samples or systematic differences that may include RNA degradation levels, read length or sequencing depth.

### cis-eQTL identification and mediation analysis

To identify the direction of effect between associated nuclear gene expression values and mitochondrial RNA cleavage rates, we carried out mediation analysis. First, we used PLINK to identify cis-eQTLs within 1MB of the start and end for each of the genes identified as significant in the comparison of nuclear gene expression and mitochondrial RNA cleavage rates using the CARTaGENE dataset, including 5 study-specific genetic PCs and 10 PEER factors as covariates. We then selected the SNP with the lowest *P*-value as a representative *cis*-eQTL and only used associations that were significant after correction for multiple tests (FDR 5%). For nuclear genes/genetic variants that pass these criteria, we tested whether the expression of the nuclear gene significantly mediated the relationship between the peak nuclear variant and associated inferred mitochondrial RNA cleavage ratios using 1000 bootstrapping simulations with the ‘Mediation’ package in R, correcting the *P*-value for the number of genes tested using Bonferroni correction. To test whether nuclear genes might be influencing mitochondrial-encoded gene expression levels through mitochondrial RNA cleavage, we obtained all mitochondrial RNA cleavage sites that were significantly associated with the expression levels of both a nuclear- and mitochondrial-encoded gene (criteria outlined above, 125 cases in total) and then performed mediation analysis by first testing whether the nuclear- and mitochondrial-encoded genes were correlated in discovery data (*P* < 0.05) and then second whether this relationship is significantly mediated by the inferred cleavage rate of the associated site in discovery data (using 100,000 bootstrapping simulations, correcting *P-*values for multiple tests).

### Gene knock down analysis

We sought knock down (KD) data for any gene that has been implicated in mitochondrial RNA processing in our analyses, including those linked through quantitative trait loci mapping (four unique genes) and expression correlation analyses (43 unique genes, Supplementary Table 5). In total, two of these genes (*TBRG4* and *RPS19*) had RNA sequencing data available after shRNA knock down (KD) as part of the ENCODE project. For each gene, there were 8 RNA sequencing datasets available in total (4 from KD and 4 from controls) in 2 different cell lines (HepG2 and K562). We obtained raw RNA sequencing datasets for each sample via the ENCODE portal (for accession numbers, see Supplementary Table 7) and then performed sequence alignment and filtering as described above. We then calculated the cleavage ratio (as above) at mitochondrial sites linked to the discovery of each gene and compared control and shRNA KD data using a one-tailed *t*-test.

### Gene enrichment analysis

Gene enrichment analysis was performed within the ‘gprofiler2’ R package [41]. The query list contained nuclear genes whose expression correlated with mitochondrial RNA cleavage rates across both discovery and replication cohorts (43 unique genes). The background gene set was defined as all unique nuclear genes tested for association with mitochondrial RNA cleavage rates across the discovery and replication datasets. The gene set counts and sizes (g:SCS) framework was used for multiple testing correction.

## Supplementary Information

**Additional file 1: Figure S1** - Distributions of putative cleavage ratios. **Figure S2** – Distributions of putative cleavage ratios at known gene boundaries. **Figure S3** – Flow chart showing cleavage site detection and validation across datasets. **Figure S4** - Correlations between inferred cleavage levels and mitochondrial-encoded gene expression levels. **Figure S5** - QQ plots for significant associations between nuclear encoded genetic variant and mitochondrial RNA cleavage rates. **Figure S6** - Association betas for discovery and replication data for associations between inferred cleavage rates at high confidence sites and common nuclear genetic variation. **Figure S7** - *P*-value distributions for association between mitochondrial RNA cleavage rates and nuclear encoded gene expression levels.

**Additional file 2: Table S1** - Inferred cleavage sites detected in CARTaGENE data. **Table S2** - Inferred cleavage sites and mitochondrial encoded gene pairs that show significant associations in both the discovery and replication datasets. **Table S3** - Significant associations in the discovery dataset between inferred cleavage rates in the mitochondrial genome and nuclear encoded gene expression. **Table S4** - Significant associations in the replication dataset between inferred cleavage rates in the mitochondrial genome and nuclear encoded gene expression. **Table S5** - Significant associations between inferred cleavage rates in the mitochondrial genome and nuclear encoded gene expression. **Table S6** - Mediation analysis for mitochondrial RNA cleavage positions. **Table S7** - Accession numbers for RNA sequencing data from shRNA gene knock (KD) down experiments in the ENCODE portal.

## Data Availability

All data generated or analysed during this study are included in this published article, its supplementary information files and publicly available repositories. RNA sequencing and genotyping data for 799 individuals obtained from the CARTaGENE project [55] was obtained through application to the data access committee (instructions are available at www.cartagene.qc.ca). RNA sequencing data from 364 individuals, along with accompanying genotyping data (obtained from either Illumina Omni5M and Omni2.5M arrays) from the GTEx project [56], was obtained by application to dbGaP through accession number phs000424.v6.p1 and is available via the GTEx portal (www.gtexportal.org). Ribosomal RNA-depleted RNA sequencing from 16 samples [29] was obtained from the Gene Expression Omnibus (GEO accession GSE136371) [65]. Oxford Nanopore sequencing data was obtained from the Nanopore WGS consortium [30] (https://github.com/nanopore-wgs-consortium/NA12878). Code and analysed data used in this work can be obtained from https://github.com/gcarbajosa/Mitochondrial_RNA_Cleavage or Zenodo [66].
