# Supplementary material for: Identification of human mitochondrial RNA cleavage sites and candidate RNA processing factors
Source: BMC Biol. 2022 Jul 22;20:168. doi: 10.1186/s12915-022-01373-5 (PMC9308231; doi:10.1186/s12915-022-01373-5)
Supplement: Supplementary file 1 — Additional file 1: Figure S1 - Distributions of putative cleavage ratios. Figure S2 – Distributions of putative cleavage ratios at known gene boundaries. Figure S3 – Flow chart showing cleavage site detection and validation across datasets. Figure S4 - Correlations between inferred cleavage levels and mitochondrial-encoded gene expression levels. Figure S5 - QQ plots for significant associations between nuclear encoded genetic variant and mitochondrial RNA cleavage rates. Figure S6 - Association betas for discovery and replication data for associations between inferred cleavage rates at high confidence sites and common nuclear genetic variation. Figure S7 - P-value distributions for association between mitochondrial RNA cleavage rates and nuclear encoded gene expression levels. [file 12915_2022_1373_MOESM1_ESM.docx]

Figure S1: Distributions of putative cleavage ratios in a) Discovery (N=799 individuals) and b) Replication dataset (N=344). Vertical red line shown at 0.1 ratio.

Figure S2: Distributions of putative cleavage ratios at known gene boundaries, as well as 100 randomly selected sites >50bp away from known gene boundaries (N=799 for each boxplot).

Figure S3: Flow chart showing cleavage site detection and validation across datasets.


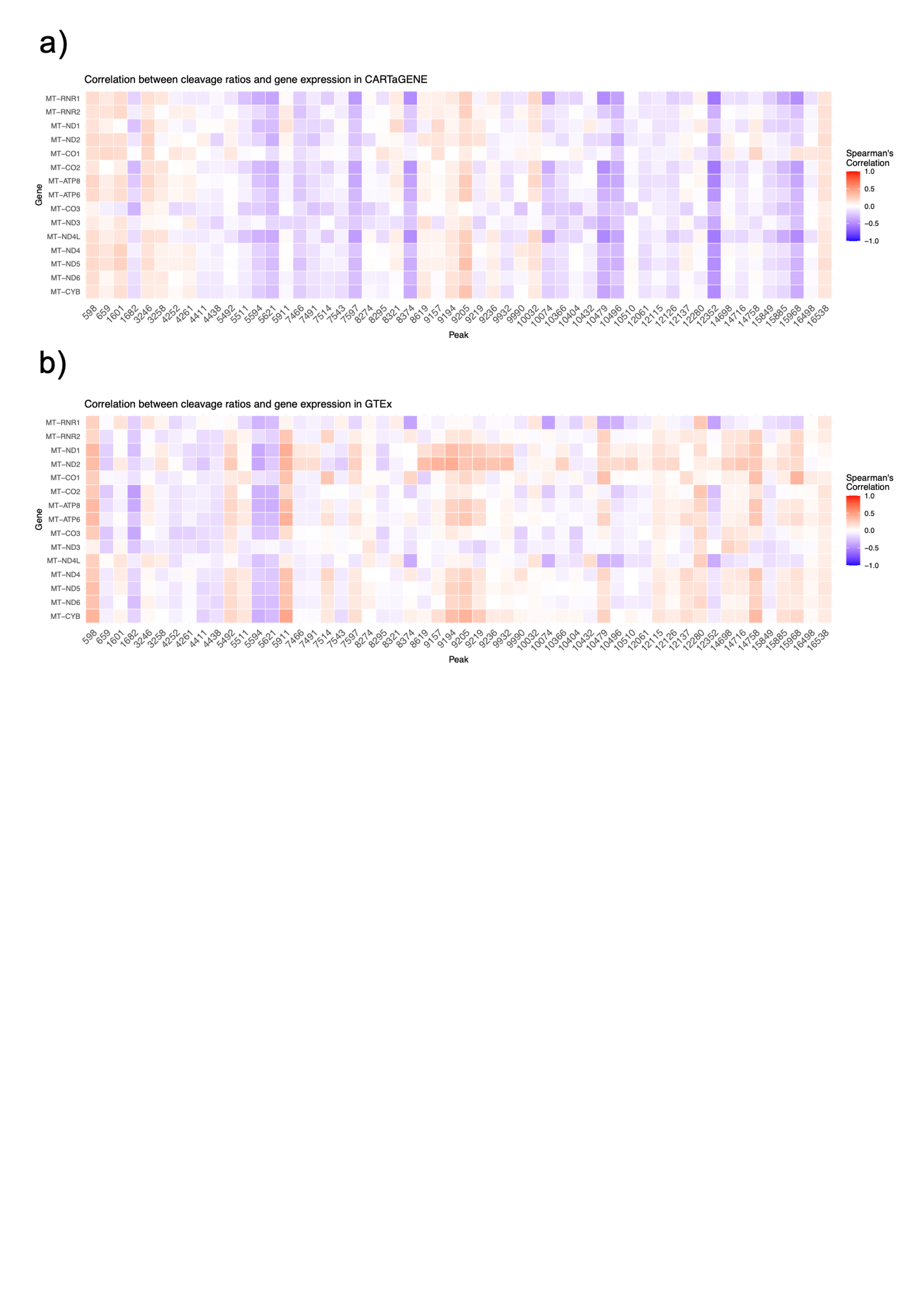


Figure S4: Spearman Rank correlation coefficients for relationships between inferred cleavage levels at high confidence sites and mitochondrial-encoded gene expression in a) discovery (CARTaGENE) data (N=799) and b) replication (GTEx) data (N=344).

Figure S5: QQ plots for significant associations between nuclear encoded genetic variant and mitochondrial RNA cleavage rates in discovery data (CARTaGENE, N=799).


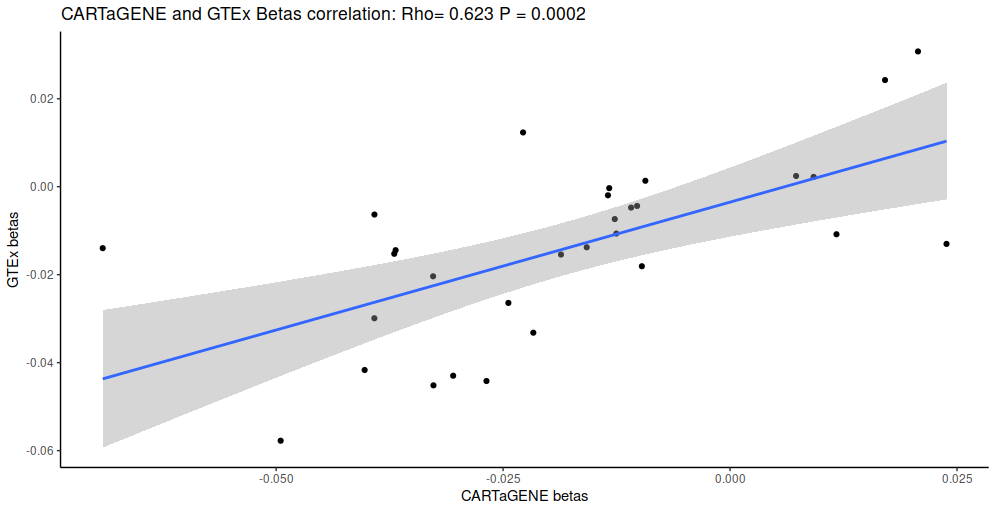


Figure S6: Association betas for discovery (CARTaGENE, N=799) and replication (GTEx, N=344) data for associations between inferred cleavage rates at high confidence sites and common nuclear genetic variation. Data points shown are for associations that reach significance after Bonferroni correction in discovery data.

Figure S7: P-value distributions for association between mitochondrial RNA cleavage rates and nuclear encoded gene expression levels in a) the discovery cohort (CARTaGENE, N=799) and b) the replication cohort (GTEx, N=344).
